# A Plan for Raising a Fund for Aged and Unfortunate Dentists

**Published:** 1911-10-15

**Authors:** L. G. Noel

**Affiliations:** Nashville, Tenn.


### A PLAN FOR RAISING A FUND FOR AGED AND UNFORTUNATE DENTISTS.

BY L. G. NOEL, D.D.S., NASHVILLE, TENN.

Read before the Tennessee State Dental Association, May 25, 1911.

We hear of so many cases of distress coming to dentists, and so many appeals for financial aid are made by the friends of these unfortunates, that I suppose we have all at times felt the need of some kind of organization provided with means to take care of such cases.

At the meeting of the National Dental Association at Asheville in 1903, I endeavored to start a movement for the accumulation of a fund for this purpose. I quote the following from my address to that body:

"Provision for Aged Members.—So many instances of aged and invalid dentists seeking assistance from professional confreres have presented themselves to my notice that I have long entertained the thought that it would be a good thing to raise a fund in our Association to be used for their maintenance. I have talked with a few of our members, and all seemed to think well of it, but no one has offered a practical suggestion as to the ways and means for raising the money.

"If we could increase our membership to several thousand, we might, in the course of time, accumulate a surplus fund as the American Medical Association has done. It is said that it has a surplus of $60,000 in the treasury.

"Do we need to wait so long for the accomplishment of an object so desirable and so beneficent?

"I think a fund for this purpose could be started by subscription. When the profession wanted money to fight frauds, it was promptly raised, and nobody who gave to the cause ever missed the contribution.

"If I could succeed in elisting your interest in this cause, you would raise $20,000 before this meeting adjourns.

"I would suggest that a Committee be appointed to solicit subscriptions to this fund, and that the Chairman of this Committee be the Treasurer, and that he be required to give a bond for the safety of the fund.

"This Committee should be ever active and diligent in soliciting funds for this object. Each State Society should be encouraged to appoint a Committee to prosecute the work, and the funds raised in the State Societies should be paid annually, or oftener, into the hands of the Treasurer of the parent committee.

"It should be the policy to invest this money in safe and reliable securities and use only the interest in alleviating the wants of those aged members applying for our charity."

I will not quote further, but will say that the idea seemed to be most heartily approved by the Association,—however my plan not being well matured, and probably because it was not a practical one, nothing was done.

It met the fate of many preceding Presidential suggestions,—died—and was completely buried in the memories of the members of the Association.

The last year has produced its crop of misfortunes, just as in the past, and while some of these are thrilling and vibrating our chords of sympathy, and at the very moment our friends are asking aid for our beloved unfortunates, I have determined to resuscitate my dormant idea, and this time I feel that I shall be able to breathe into it a life that shall persist, grow, develop and bear fruit.

This time I propose a plan that is practical, simple and sensible. Instead of starting in the head or top of the tree, I shall start with the roots; instead of in the National Dental Association, I propose to start the movement in the State Associations; and my plan is as follows:

Let each State Society increase the annual dues $1.00 for each member, and let the additional $1.00 thus obtained be turned over to a Committee to be known as the Benevolent Committee.

I would suggest that this Committee should consist of three members, and that they be elected by ballot, the senior one to serve three years, and the junior members to serve one and two years respectively, so that at each annual election there will be one place to fill on this Committee.

It shall be the duty of this Committee to take care of the Benevolent Fund, to solicit subscriptions to it whenever and wherever in their judgment they are likely to be obtained, to receive any donations or legacies that may come to their care, and to report to a parent Committee in the National Dental Association any cases of distress among our members, that in their judgment, are in need of and deserving our attention; paying over annually to the parent Committee the money collected during the year.

This plan will be carried up to the National Dental Association at Cleveland, and if it meets with the approval of that body, a parent Committee will be elected, whose duty it will be to act in the capacity of parent Benevolent Committee and push the organization of this work in all State Socities (in local Societies as well if they choose to enter upon the work), until all become co-laborers and sharers in the benefits of this organization.

Local Societies entering this work are to make annual reports and payments of funds into the hands of the State Committees; and these in turn are to make annual report to the National Committee; which latter Committee is to be placed under bond for the safeguarding of the fund.

Now, let us see how the fund may be expected to grow, if we complete the organization and collect $1.00 a year from each of our members.

There are about 47 States. If each State organizes a Dental Society, with the populous Easterm and Northern Socieities of long standing, many of which now number several hundred members, I believe it is safe to say we shall soon average 100 members for each State Association. This would give us 4700 members, worth as many dollars annually to our benevolent fund.

This fund if wisely husbanded should grow from year to year into a very strong bank, yielding a goodly interest to take care of our unfortunates.

The methods of the parent Committee in managing the money should be such as are followed by Insurance Companies and such like Benevolent organizations; in short, methods that are safe, sane and yielding such return of interest as may be realized from safe investments.

It is my earnest belief that such an organization as I have outlined will add more members to our State and Local Societies and to our National Association than anything else we can do.

If we put enthusiasm into our efforts and honest work behind our enthusiasm, there is no telling what we may do in this line. Success once assured begets more success. A goodly fund once established will grow very rapidly, and wise management will be sure, in the course of time, to attract some considerable legacies; but we need not indulge in dreams like Alnascar's to arrive at figures well calculated to stimulate us to our best efforts.

I do not offer the above plan as a perfect one,—perfection is seldom attained, and in efforts like this can only be reached, if ever, after years of trial.

It is my hope that some of you who have business ability will promptly suggest improvements. At least, I hope you will think well enough of the movement to accept it as one worthy of our best efforts, and that you will regard it as a duty that rests upon us as individuals, as well as in our associated capacity.

DISCUSSION.

Dr. H. W. Morgan: Accompanying this paper, Mr. President, is a letter from a prominent dentist in the West, appealing to Dr. Noel for a contribution for one of the largest contributors to dental literature upon the subject of dental anatomy and comparative anatomy, who is financially in distress as the result of a stroke of paralysis, I believe. His home is mortgaged, and recently this matter has been made the subject of a canvas in the city of Nashville, and Dr. Noel has obtained some right encouraging contributions for the relief and removal of the mortgage that is on the home of this distinguished Kansas dentist. He asked me to say to you that he hoped you would take this matter up and do something in the way of a permanent organization, whether you attempt to outline the organization now or merely adopt resolutions committing yourselves to some plan by which in the future it may be taken up and perfected is immaterial to him, but he does earnestly hope that you will place your stamp of approval on it, and that your hearts will go out to these unfortunates in our ranks who are not able to take care of themselves. The gentlemen who collected the largest dental fee that was ever paid for a single filling on American soil was buried by the charity of his brethren. There is not a man sitting before me now but in the course of the next few months or years, may have everything he has on earth swept away and stand in the same position as this brother in the West, whose case appeals so earnestly to us. I have known more than one dentist who owed his brethern the bread that he ate during the last five years of his life and I believe some plan should be instituted to take care of these deserving and suffering dentists in their old age.

Dr. Neil: Mr. President, I move you that a committee of three be appointed, with Dr. Noel as chairman of the committee, and two others in connection with him, to take this matter up and put it into active force.

Motion seconded.

Dr. Beech: If this is to be done in a business like manner and this is to be a permanent organization committee, it occurs to me that the proper thing to do is to even so amend the constitution so that this committee may be a standing committee like the other standing committees, stating its duties; also that the members of the committee are elected by ballot at this meeting, serving respectively one, two and three years, and let this committee get to work as an official committee from the State Association. It undoubtedly is a worthy cause and should be done. There is not a man here to-day but what, as Dr. Morgan has stated, may in a very short space of time, of necessity have to call upon his professional brothers, or upon his close friends—and there should be none closer than a professional brother—for his personal support. I believe if the Doctor will accept the amendment, that more good can be accomplished for the simple reason that committees are appointed by the Association to do certain work and the committees fail to do their work, they are not like the executive committee, or membership committee, or other committees that are elected by this body and not appointed by the president, therefore if the gentleman will accept the amendment I would amend the motion as stated by Dr. Neil.

The President: I suggest that this amendment to the constitution must be put in writing. As I understand the motion at the present time, the constitution and by-laws are to be so amended that we have a standing benevolent committee composed of three members elected by the Association, one member to be elected each year.

The motion as amended was passed unanimously.

